# Observing the earth from space: Does a virtual reality overview effect experience increase pro-environmental behaviour?

**DOI:** 10.1371/journal.pone.0299883

**Published:** 2024-05-29

**Authors:** Femke van Horen, Marijn H. C. Meijers, Yerong Zhang, Michael Delaney, Annahita Nezami, Paul A. M. Van Lange

**Affiliations:** 1 Marketing Department, School of Business and Economics, Vrije Universiteit Amsterdam, Amsterdam, The Netherlands; 2 Communication Science Department, Faculty of Social and Behavioural Sciences, Universiteit van Amsterdam, Amsterdam, The Netherlands; 3 Human Factors and Settlement Department, Kepler Space Institute, Bradenton, Florida, United States of America; 4 Royal Society of Arts, Manufactures and Commerce, London, United Kingdom; 5 Department of Experimental and Applied Psychology, Faculty of Behavioral and Movement Science, Vrije Universiteit Amsterdam, Amsterdam, The Netherlands; University of Sheffield, UNITED KINGDOM

## Abstract

Astronauts (and recently businessmen) often express a renewed sense of responsibility for taking care of the environment, after observing the overwhelming beauty of Earth from space. Despite recent attention for this “overview effect”, it is unclear whether experiencing the effect directly impacts pro-environmental behaviour. Using a virtual reality experience, the current research tests in two experimental studies the direct impact of an immersive overview effect experience on both short-term and longer term subsequent pro-environmental behaviours (donating to an environmental NGO, consuming less diary and meat). Furthermore, it investigates whether the technological immersiveness of the VR experience amplifies the effect, and the mediating role of connectedness to nature. Results show no effects of the short (7 minutes) overview effect VR video on pro-environmental behaviour (Study 1). For the longer video (15 minutes, Study 2), the results showed that the most immersive experience (video featuring meditative music and voice-over) appeared to increase connection with nature and higher donation amounts to an eco-NGO, but not significantly. No effects were found for subsequent meat and dairy consumption behaviours (measured on day 2, 4, and 6). These findings contribute to a deeper understanding of the specific features determining the effectiveness of the overview effect experiences on actual pro-environmental behaviour, providing important insights to businesses and educational institutions.

## Introduction

After returning from space, astronauts report how amazed and awestruck they are by Earth’s beauty and fragility after which they report a stronger emotional connection with nature. This experience is referred to as the “overview effect”, which is characterized by powerful emotions and feelings of connectedness with humankind and the planet as a whole [1–3]. Many astronauts, such as Dutch astronauts Wubbo Ockels and André Kuipers, and American astronauts Nicole Stott and Ronald Garan, devoted their life to sustainability and pro-environmental action after they returned home. More recently, even businessmen such as Jeff Bezos experienced during his spaceflight these feelings of beauty and wonder for planet Earth. Upon his return he realized that he needed to do more to preserve the Earth and donated $10 billion dollars to help fight climate change.

The potential of the overview effect to spur on pro-environmental behaviour has been reported frequently in the media and inspired educational programs and museum exhibitions. For example, at COP26 in Glasgow, a replica of the Earth was installed by an artist to give visitors a hint of the overview effect [4]. Furthermore, several museums provide visitors a unique, extra-terrestrial experience of the Earth through large video installations to “forever change the way visitors look at our planet” (e.g., Discovery Museum, https://www.discoverymuseum.nl/en/activities/earth-theatre/). The non-profit foundation Spacebuzz (https://www.spacebuzz.earth/) has developed a virtual reality (VR) assisted educational program for children based on the overview effect which aims to make children ambassadors of planet Earth. Despite the recent attention surrounding the overview effect and its potential positive effects on pro-environmental attitudes and behaviour, it has so far never been systematically tested whether experiencing the overview effect can indeed directly impact people’s pro-environmental behaviour.

The current research investigates this gap and has three goals: First, we will examine whether experiencing aspects of the overview effect using immersive technologies (i.e., virtual reality) can predict short-term and longer term pro-environmental behaviour (e.g., donating to an environmental NGO’s, consuming less diary and meat). Second, as spaceflight is not accessible for many people and is in fact detrimental to the environment, we attempt to simulate the overview effect experience using VR, a powerful tool to induce a sense of spatial presence (e.g, [5, 6]). We will test the effectiveness of the virtual reality experience of the overview effect by systematically varying its level of immersiveness. Finally, we will study connectedness with nature as a potential underlying mechanism [7, 8]. Previous studies have shown that connectedness with nature is strongly associated with pro-environmental behaviour [9, 10] and the sense of connectedness is key when experiencing the overview effect [1, 11]. Whereas exploratory studies have shown that a VR overview effect experience can reliably induce awe [12–14], its effects on a sense of unity and connectedness are understudied [14], and its effects on pro-environmental behaviour change await to be examined.

This research will add to the limited and predominantly qualitative research on how the overview effect may increase actual pro-environmental behaviour [11, 15] and it will provide a deeper understanding on the efficacy of (educational) programs using a VR overview effect experience. Establishing the effectiveness of the overview effect is important as it would allow us to create interventions for businesses and public education campaigns to increase environmental awareness–and action–among children, managers, and society at large.

### Overview effect

The overview effect is a phenomenon first coined by the space philosopher Frank White to describe the psychological experience reported by astronauts as a result of seeing planet Earth from the perspective of space [2]. The profound emotional experience is triggered when astronauts are Earthgazing and see Earth as a tiny blue ball floating in a vast universe, astounded by its aesthetic beauty and sublimity. At the same time, they realize Earth’s vulnerability due to the thinness of the atmosphere that protects Earth and the anthropogenic (man-made) destruction of Earth (e.g., forest fires, [11]). Astronaut reports and interviews suggest that during Earthgazing familiar points of interest (such as observing their country of birth or residence) are notable, however, observing the Earth as an interconnected and unified whole, instead of separated countries, is more emotive and optimizes the overall experience [1, 3, 11]. One astronaut, for example, described the overview effect as an “overwhelming sense of oneness and connectedness accompanied by an ecstasy… an epiphany” ([16], p. 73). The psychological state of the overview effect thus seems to consist of two core elements: a profound emotional experience and the feeling of connectedness to Earth.

Research on the overview effect is scarce, predominantly consisting of exploratory, qualitative studies with small samples of astronauts to define its features [1, 11, 15]. Other research has focused on the emotional experience of the overview effect, with a particular focus on feelings of awe and self-transcendence [3, 17]. For instance, it has been shown that a VR experience of the overview effect elicited an awe-inducing experience [14] and that for children high in dispositional awe the VR-induced overview effect was positively associated with their learning performance in the domain of nature conservation [18].

The influence of the overview effect on both people’s sense of unity and connectedness with the Earth and their actual pro-environmental behaviour is however largely unexplored [14]. The evidence consists of one qualitative study in which astronauts who experienced the overview effect were interviewed [11]. The astronauts reported higher positive attitudes toward nature and a greater concern for environmental issues. They also mentioned an increase in their personal pro-environmental behaviours like adopting a more sustainable diet, using reusable paper bags for groceries, and decreasing water usage. However, to date, very limited research exists that systematically investigated the causal effect of the overview effect on pro-environmental behaviour. To our knowledge, only Chirico and colleagues [19] in a recent study, showed that virtual nature exposure in combination with awe affects pro-environmental behaviour (number of flyers about plastic pollution taken) as compared to a control condition, but unexpectedly, an awe-inducing, non-nature overview effect experience did not. In sum, so far it is unclear whether the overview effect experience can stimulate connectedness to nature as well as pro-environmental behaviour. Therefore, adding to the limited (primarily qualitative and correlational) literature, the main goal of the current research is to investigate the effectiveness of the overview effect directly and systematically with different levels of immersiveness on both short and longer term pro-environmental behaviour.

### Connectedness to nature

There are two possible explanations as to why people are inclined to perceive more connectedness to nature when experiencing the overview effect. First, as mentioned above, directly seeing the Earth from space as a coherent ecosystem leads astronauts to further contemplate about life on the planet and to an increased feeling of connectedness with Earth, including humankind and nature [1, 3, 11, 15]. We posit that the overview effect experience increases people’s perceived connectedness with nature, or the degree to which people view themselves as part of the surrounding natural environment [20–22]. The most common experience that increases a sense of connectedness with nature, is direct contact with nature [23, 24]. However, self-transcendent experiences not involving direct contact with nature, such as meditation or the use of psychedelics, can also heighten the sense of connectedness with nature [25, 26]. We therefore posit that the overview effect may, aside from engendering a feeling of self-transcendence [3, 17], also instigate a stronger sense of connectedness to nature.

Second and related, when observing the Earth from a distance, “zooming out and seeing the bigger picture” may enhance a sense of nature connectedness. To increase connectedness to nature, research has predominantly focused on how the natural world can be brought closer, for example, by taking the perspective of an animal being harmed by pollution [8, 27]. In addition, as environmental issues are often perceived by people as abstract and future oriented, research has mostly concentrated on how environmental challenges and benefits should be made more concrete to change behaviour [28]. For instance, research has demonstrated that emphasizing the immediate, rather than longer term benefits of sustainable products, increased people’s willingness to purchase these products [29]. Here, we posit that not only by ‘zooming in’ climate problems can become more concrete and closer to the self, but also by ‘zooming out’ from the natural world. By zooming out, people might realize they are a part of nature and the Earth they belong to, which can lead to a stronger sense of connectedness to nature.

### Pro-environmental behaviour

People’s food consumptions decisions have a significant impact on the environment: food choices do not only account for 25% of the CO_2_ emissions, but also importantly affect unsustainable land usage, decrease in biodiversity, and freshwater shortage [30]. Thus, to really make a difference it is of importance to study how food decision-making and choices can become systematically more pro-environmental. Previous literature has demonstrated that connectedness to nature is not only generally associated with the intention to behave more sustainably [24] (for a recent meta-analysis see [31]), but also to more specific food choices and donation decisions. For instance, it has been shown that when people are highly connected to nature, they also tend to choose more often vegan and vegetarian options [32] and are more willing to donate to environmental causes [33]. Based on these findings, we investigate in the current study the effects of the overview effect experience on connectedness to nature, dietary behaviour and donations, and test whether connectedness to nature mediates the effects.

### Virtual reality as immersive technology

To test whether experiencing the “overview effect” will foster pro-environmental behaviour through increased connectedness with nature, we will use virtual reality (VR). VR can be a powerful instrument to simulate experiences that are poorly accessible in the real world (such as spaceflights) in an ecologically valid way. As compared to, for instance, a 2D video or a vignette study, a VR experience is high in realism as it has the potential to mimic the actual experience of Earthgazing from the space perspective as closely as possible, while simultaneously maintaining high experimental control [34]. VR is effective when it elicits a sense of spatial presence, meaning that people feel present in the environment being presented [5, 6], which can create the illusion of a direct environmental experience [35]. Thus, by using VR to induce the overview effect, people might feel they are in space, orbiting around our Earth while being in the laboratory. Indeed, exploratory studies have shown that VR can be used to elicit the overview effect experience and can reliably induce awe [13, 19]. Here, we test whether a VR overview effect experience can lead to a stronger connectedness to nature, and subsequently to increased pro-environmental behaviour.

### The current research

To investigate these questions, two experimental studies were set up. In the first controlled experiment in which a 7-minute video of the overview effect was created, we investigate (1) whether experiencing the overview effect (as compared to the two control conditions) increases people’s intentions to buy biological food and their donation amount to a pro-environmental organization, and (2) whether connectedness to nature mediates the effect on donation. For the second experiment, we created an elongated 15-minute video of the overview effect and three different levels of technological immersiveness: 1) visual experience of the extraordinary and expansive landscape (as in Study 1), 2) transcendent music and 3) meditative narrative. We test the effect of the overview effect video with different levels of immersiveness on actual donation amount to a pro-environmental cause, as well as the follow-up effects by investigating consumers’ meat and dairy consumption at different points in time. For both studies, we collaborated with EarthscapeVR (www.earthscapevr.com) who specialize in creating evidence-based VR-assisted courses and workshops that capture the essence of the overview effect with the aim of promoting mental health and sustainable behaviours.

The current research seeks to contribute to the existing literature in several ways. First, to our knowledge, this project is the first to systematically test the impact of the overview effect experience on short-term and subsequent pro-environmental consumer behaviour in a controlled experimental setting. Second, the studies can give further insights on whether VR is a suitable tool for eliciting the overview effect and whether it can be used to influence pro-environmental behaviour in a similar way to actual space travel [11], but less detrimental to the environment. Third, we test different versions of the VR experience to optimize the potential VR overview effect. For example, we examine how the level of immersion (through music and inclusion of a voice-over) influences the effectiveness of the VR experience on people’s connectedness to nature and their pro-environmental behaviour. These insights are of importance so that an effective overview effect experience can be developed, which can be used for interventions for children at schools, for businesses, and for the general public to create more environmental awareness. Fourth, our research adds to the limited body of research that studies longer lasting effects of VR on pro-environmental behaviour, providing more insights into the potential of VR for stimulating (pro-environmental) behaviour change (e.g., [35, 36]). Lastly, the current studies add to the more common ‘zooming in’ approach (focusing on how to “frame” environmental impact in concrete terms) by testing whether ‘zooming out’ and looking at the bigger picture by means of virtual reality could elicit a sense of connectedness to nature and pro-environmental behaviour.

## Study 1

### Method

#### Participants and design

An a-priori power analysis (G*Power, [37]) revealed that a target sample size of 159 was needed to detect a medium effect size of *f* = 0.25 with sufficient power (1-β > 0.80, α = 0.05). Final sample size was determined by availability of subjects at the university’s behavioural lab.

Hundred and fifty-five students from a large European university were recruited in March 2022 and randomly assigned to one of the three conditions (VR overview effect [VR OE], VR control [VR C], no-VR control [C]). We excluded 10 participants: nine failed the attention check and one did not partake in the manipulation but immediately started the survey, resulting in *N* = 145 for final analyses (*n*_VR OE_ = 51, *n*_VR C_ = 48, *n*_C_ = 46; 118 female, 1 non-binary; *M*_age_ = 20.57, SD = 1.65). All participants gave informed consent for study participation and received course credits as compensation for their participation. The study was approved by the Ethical Review Board of the University [Anonymized for review] (ERB number: 2022-PC-14663). Authors did not have access to information that could identify individual participants during or after data collection. The experimental materials, statistical analyses codes, and data of Studies 1 and 2 can be found at OSF (https://osf.io/5yg72/). All implemented experimental conditions are reported and all measured variables are disclosed.

#### Materials

**Fig 1:**
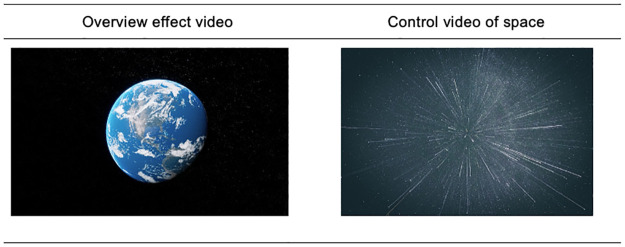
Screenshot from the VR overview effect video and still of the VR control video Left: Reprinted from Earthscape VR under a CC BY license, with permission from Charles Perring, original copyright 2023; Right: Still of the VR control video. *Note*. There are some slight differences between the video used in the study and the picture of the control video presented here due to copyright issues (designed by Freepik, freepik.com).

In the VR overview effect condition, participants experienced the overview effect through VR, observing the Earth from space. Participants were shown an edit of 7 minutes from the original 30 minutes 180° VR overview effect experience created by EarthscapeVR^®^ via a head-mounted display (i.e., Pico G2 4K). This video was created based on the NASA blue marble dataset and scientific research on the overview effect [1]. In the 7-minute edit participants first saw the Earth rise from behind the Moon. They then slowly travelled through space from observing Earth from orbit, across Europe and South America while darkness fell over Earth, finally zooming out until Earth disappeared in space. Not to distract the participants, we chose for a non-interactive approach. However, participants were able to move their head and body allowing them to observe the full 180° video. In the VR control condition, participants experienced space travel through the stars without observing the Earth. The video used in this condition was a 180 VR experience found on YouTube (https://www.youtube.com/watch?v=zBpLGfeSHAE) which was also edited into a 7-minutes version (Fig 1). A VR-control condition was added, as simply undergoing a virtual reality experience may have an impact on participants in other ways (e.g., interest, positive affect, involvement) aside to the overview effect.

In the no-VR control condition, participants were instructed to fill out a set of filler tasks on paper for the same duration as the VR conditions. The tasks were space-related quizzes and a space-themed word search puzzle. Additionally, the filler tasks were designed to take longer than seven minutes, so that the researchers could stop the participant at the seven-minute mark, ensuring the control group spent the same time as the participants who watched the VR videos. In all three conditions a white noise sound was played over the headphones either during the VR experiences or while working on the filler task, to control for sound as a potential confound.

#### Procedure and measures

Participants entered the lab, provided informed consent, and were placed in a cubicle with a computer. They were instructed to stay seated throughout the experiment. All participants were first asked to read a short article about the planetary boundaries [38]; see S1 Appendix in S1 File, to make the topic equally top of mind for all participants before starting the experiment and to guide the experience. In the two VR conditions participants were asked to put on their VR headset after giving them a brief user instruction. In all conditions, participants were then asked to put on the headphones with white noise during the entire 7-minutes of the VR experience/filler task. After the 7-minutes, the participants were instructed to take off the VR equipment or stop the filler task, and to take off the white noise headphones. They then proceeded with the survey presented on the computers.

First, we measured participants’ perceived spatial presence during the VR experience with the self-location subscale from the Spatial Presence Experience Scale (SPES, [39]), consisting of a four-item scale (e.g., “I felt like I was actually there in space”, α = .94; *M* = 3.76, *SD* = 1.68) ranging from 1 (*strongly disagree*) to 7 (*strongly agree*; see S 2 for all measures and scale items of Studies 1 and 2). To measure people’s connectedness to nature, we adapted the Inclusion with Nature in the Self scale [40]. This is a pictorial measure composed of diagrams, each representing different degrees of overlap of two circles representing the self and nature. The overlap progressed linearly in seven steps starting with 1 (*no overlap*) and ending with 7 (*almost total overlap*), indicating high connectedness with nature. We adapted the scale by replacing “Nature” with “Earth” to measure participants connectedness to the Earth more directly.

To examine participants’ pro-environmental behaviour, they were asked to take part in a shopping task and a donation task (adapted from [29]). In the shopping task participants were randomly presented with a total of 20 products to choose from, consisting of 10 product categories (apples, cheese, etc.) each with two choice alternatives–a conventional and a sustainable option–presented next to each other (counterbalanced) accompanied with its actual price (e.g., conventional potatoes 3kg €3,99 and sustainable potatoes 3kg €4,89). Participants were then asked to select 5 out of the 20 products that they wanted to buy. They were told that they have enough money to spend on any combination of groceries, to avoid financial constraints affecting their decision-making. The number of sustainable products put into their shopping basket was the dependent variable.

We used an incentive compatible donation task to assess participants’ willingness to donate to an environmental organization (the National Society for Nature Conservation). Participants were informed that four students would be randomly selected who would win €25 after completion of the study. They were then asked, if they would win the €25, how much of this money they would like to keep for themselves and how much they would be willing to donate to the National Society for Nature Conservation. After data collection, the amount the winners of the lottery had indicated to donate (vs keep) was indeed donated to the organization (vs. to the winners, [29]).

Participants were then asked to complete a six-item scale to assess dispositional awe as an exploratory measure (subscale from the Dispositional Positive Emotion Scales [41], α = .73; see S2 Appendix in S1 File). As an attention check, they were asked to ignore the next question (“How likely are you to travel with an airplane?”) and to simply drag the slider to 40. Finally, cybersickness was assessed by indicating whether they experienced nausea during the space experience (*yes/no*). At the end of the survey, participants completed some demographic questions (age, gender) and were thanked for their participation. See S3 Appendix in S1 File for correlations across all variables.

### Results

#### Spatial presence

A one-way ANOVA revealed that the manipulation was successful, as there was a significant difference in spatial presence ratings across the three conditions (*F*(2, 142) = 40.51, *p* < .001, η^2^ = .36). Planned contrasts showed additionally that both the VR OE condition (*M* = 4.69; *SD* = 1.39) and the VR C condition (*M*_VR C_ = 4.16; *SD* = 1.32) induced an increased sense of spatial presence as compared to the no-VR control condition (*M* = 2.32; *SD* = 1.34; VR OE: *F*(1, 142) = 74.78, *p* < .001, η^2^ = .35; VR C: *F*(1, 142) = 43.79, *p* < .001, η^2^ = .24, respectively). The difference between the two VR conditions was as expected, but statistically not significant (*F*(1, 142) = 3.82, *p* = .053, η^2^ = .03).

#### Connectedness to nature

To test whether the VR overview effect experience had an impact on participants’ sense of connectedness to the Earth, a one-way ANOVA was conducted. The results revealed no differences between conditions (*M*_VR OE_ = 4.57, *SD* = 1.49; *M*_VR C_ = 4.71, *SD* = 1.34; *M*_C_ = 4.72, *SD* = 1.28; *F*(2, 142) = 0.18, *p* = .833, η^2^ = .003). The hypothesis that the VR overview effect would significantly heighten one’s sense of connectedness to the Earth, was not supported by these results.

#### Shopping task

To test whether the overview effect experience increased sustainable consumption behaviour, we conducted a one-way ANOVA. The results showed no significant difference across the three conditions in the number of biological items put in the shopping basket (*M*_VR OE_ = 3.24, *SD* = 1.64; *M*_VR C_ = 3.35, *SD* = 1.64; *M*_C_ = 3.39, *SD* = 1.48; *F*(2, 142) = 0.13, *p* = .880, η^2^ = .002).

#### Donation amount

The results of a one-way ANOVA revealed that there was no significant difference between the conditions in the amount they were willing to donate to the National Society for Nature Conservation (*M*_VR OE_ = 13.75, *SD* = 8.79; *M*_VR C_ = 15.01, *SD* = 8.20; *M*_C_ = 14.36, *SD* = 8.95; *F*(2, 142) = 0.27, *p* = .768, η^2^ = .004).

#### Mediation of connectedness to nature (INS) on donation amount

We hypothesized that the overview effect experience would raise donation amount due to an increased sense of connectedness with nature. Mediation analyses (Process multicategorical Model 4, [42]) showed however that the indirect effect via connectedness to nature was not significant (VR OE vs. VR C: point estimate .08, 95% CI [-.35 .68]; VR OE vs. C: point estimate .09, 95% CI [-.26 .77]).

#### Robustness checks

We checked whether cybersickness influenced any of the above results. Nineteen participants reported cybersickness (*N*_VR OE_ = 8, *N*_VR C_ = 10, *N*_C_ = 1). After exclusion, the results remained the same; there were no significant differences for any of the dependent variables (*F*_Connectedness_(2, 123) = 1.45, *p* = .239, η^2^ = .02; *F*_Shopping_(2, 123) = .29, *p* = .752, η^2^ = .005; *F*_Donation_(2, 123) = 1.34, *p* = .267, η^2^ = .02). See S4 Appendix in S1 File for exploratory analyses on dispositional awe.

### Discussion

The results of Study 1 did not show a significant effect of the VR overview effect on people’s connectedness to nature. In addition, there was no significant difference in pro-environmental behaviour, neither on the shopping task nor on the amount donated to an eco-NGO, when comparing the overview effect experience condition with the VR and no-VR control conditions. Finally, connectedness to nature did not mediate the effect of the VR overview effect on donation amount.

## Study 2

Study 2 was set up to extend the results of Study 1 in several important respects. First, as the non-significant results of Study 1 may be due to the very short VR video, we elongated the video from 7 to 15 minutes. Second, we included three different versions of the VR overview effect (VR OE), to test the effect of a more immersive experience (e.g., adding music and a voice-over). Third, we chose a less stringent VR control condition, this time not using a space related VR control condition, but a VR control condition showing kaleidoscopic figures. Fourth, aside to short-term pro-environmental behaviour, we also tested for longer lasting subsequent effects by investigating people’s meat and dairy consumption at three points in time. Finally, for exploratory purposes, we included several state questionnaires to investigate whether people’s sustainable intentions and environmental awareness increased after experiencing the overview effect, aside to their connectedness to nature and actual pro-environmental behaviour. The study was approved by the Ethical Review Board of the University [Anonymized for review] (ERB number: SBE5/8/2022fhn740).

### Method

#### Participants and design

An a priori power analysis revealed a target sample size of 305 to detect a small to medium effect size of *f* = 0.20 for five groups with sufficient power (1-β > .80, α = .05). As Study 2 has a longitudinal design, we accounted for a 10% attrition rate. Final sample was determined by the availability of students in the university’s lab.

Three hundred thirty-three students at a large university were recruited in May 2022 and randomly assigned to one of the five conditions (VR OE no-music, VR OE with music, VR OE with music and voice-over, VR control and no-VR control). All participants gave informed consent for participating in the study and received course credits as compensation. Participants who failed the attention check (*n* = 14), started the survey before the manipulation (*n* = 5); stopped the video/task early (*n* = 8), or did not follow lab instructions (*n* = 2) were excluded, leaving *N* = 304 for final analyses (103 female, 1 preferred not to indicate; *M*_age_ = 20.86, *SD* = 1.84).

#### Materials

In the VR OE no-music condition, the 180° VR OE video (1920 x 1080 resolution) of Study 1 was edited into a 15-minutes version. In this version participants first observed the Earth from above, after which they travelled around the globe before finally zooming out from Earth into deep space. The experience uses well-known imagery such as Earthrise (i.e., seeing Earth appear as one normally sees the moon appearing from Earth) and the Pale Blue Dot (i.e., seeing Earth from a further distance). In the VR OE music condition, music (soundscape by EarthscapeVR) was edited into the video. Finally, in the VR OE voice-over condition, in addition to the music, the voice of “Gaya” was edited into the video (meditation by EarthscapeVR), which was a female soft voice with a meditative script describing how humans are related to Earth. A Pico 2G 4K VR headset was used.

To obtain a cleaner comparison between the VR control and experimental conditions, we selected, contrary to Study 1, for the VR control condition a video with no references to space, as space travel is in essence part of the overview effect experience. Participants watched a 15 minutes 360 VR experience (1920 x 1080 resolution), showing a unicolor kaleidoscopic sphere with slowly changing patterns (https://www.youtube.com/watch?v=RvpEkf1jJqA&t=132s). In the no-VR control condition, participants were asked to read some news articles (about exercising, gadgets, sports) after which they responded to some questions, for the same duration as the VR conditions (15 minutes). In the VR OE no-music, VR control and no-VR control conditions a white noise sound was played over the headphones while going through the VR experience or while working on the filler task, to control for sound.

#### Procedure and measures

*Main experiment*. The procedure of the lab experiment was similar to Study 1. After informed consent and general instructions were given, participants were seated behind the computer and were instructed to read a short article regarding climate change, this time with a focus on ways to reduce meat and dairy consumption (see S5 Appendix in S1 File) creating a rationale for the later follow-up study. Participants in the VR conditions were then given a short instruction on the VR-equipment and were asked to put on their headphones during the 15-minutes VR experience. In the no-VR control condition participants were instructed to put on the headphones, to read the articles on the computer and to answer the questions for 15 minutes. After the first task, the participants were instructed to take off the headphones (and VR equipment depending on condition) to proceed with the, ostensibly unrelated, survey presented on the computers.

Participants were first asked, similar to Study 1, to indicate their spatial presence using the Spatial Presence Experience Scale (α = .93; [39]) and to indicate their connectedness to nature (instead of Earth) using the Inclusion with Nature into Self scale (INS, [40]). Then, to measure pro-environmental behaviour, we used the same donation task as in Study 1. After the attention check (same as in Study 1), participants were asked to fill out several questionnaires investigating sustainable intentions and environmental awareness. To measure the extent in which the experience of the VR overview effect has on people’s current sustainable intentions and environmental awareness, we changed the items of all scales into state- (Right now, I …”), rather than trait items. First, as connectedness to nature is our core concept, we added the Connectedness to Nature Scale (CNS, [7]), which focuses on the emotional component, rather than on beliefs (as Inclusion of Nature in Self scale does; [43]) and correlates highly with INS [44]. Participants were asked to indicate the extent in which they agreed to 13 statements (one item from the original scale was excluded), such as “Right now, I feel a sense of oneness with the natural world around me”; α = .85; see WA B for all items of all scales), followed by several exploratory questionnaires (Commitment to the Environment Scale (ten items, α = .88, [45]); Involvement in the Topic of Climate Change (four items, α = .86, [46]); Environmental Concern scale (five items; α = .77, [47]), all state-versions). After the individual state measures, cybersickness was measured with 6 items retrieved from the Simulator Sickness Questionnaire to assess if participants felt nausea during the study (e.g., “did you feel fatigue during the first task of the study” α = .77; [48]). For all the above measures 7-point Likert scales ranging from 1 (*strongly disagree*) to 7 (*strongly agree*) were used. At the end of the study, participants answered some demographic questions (age, gender), were asked to create a unique identifier, and were instructed that they would receive a very short follow-up questionnaire 2, 4, and 6 days after the experiment, which they were requested to answer within 24 hours.

*Follow-up questionnaires*. Participants received three follow-up questionnaires (2, 4, and 6 days after the experiment) via email each day at 8pm (anonymity of the participants was guaranteed through the usage of unique codes). To measure the subsequent effects of the VR OE experience on meat and dairy consumption, we asked participants on the second, fourth, and sixth day after the experiment, to indicate how much meat they consumed on that day (for breakfast, lunch, dinner, and in-betweens [35]) ranging from 1 (0 grams), 2 (1–75 grams), 3 (75–150) … to 6 (> 300 grams), with each step increase of 75 grams, and 7 (I never eat meat). As it is difficult to estimate how many grams of meat have been eaten, an example was given for each choice option (e.g., 75–150 gram, as big as a deck of cards, e.g., a hamburger; cf. [49]), see WA B for exact answer scales and all examples). After, they were asked how much dairy they consumed on that particular day ranging from 1 (0 grams), 2 (1–200 grams, e.g., a glass of milk), 3 (201–400, e.g., a glass of milk and two buttered sandwiches with a cheese slice) … to 6 (> 800 grams), with each step an increase of 200 grams, and 7 (I never consume dairy). Again, examples were given so that participants could give a better estimation. Finally, two measures were included for exploratory reasons: How often the participants had talked that day to friends, family, co-workers, fellow students or others, about climate change, on a 7-point scale ranging from 1 (*not at all*) to 7 (*a great deal*) and (2) the extent in which they agreed with the statement “I intended to engage in environmentally friendly behaviour today” from 1 (*strongly disagree*) to 7 (*strongly agree*), see S2 and S6 Appendices in S1 File). For correlations across all variables see S7 Appendix in S1 File.

### Results

#### Spatial presence

The results of a one-way ANOVA revealed that the manipulation was successful: the difference in presence ratings across conditions was significant (*F*(4, 299) = 7.87, *p* < .001, η^2^ = .10). Planned contrasts showed that sense of presence was, as compared to the VR control condition, significantly higher in all three VR OE conditions (*F*_VROE NM_ (1, 299) = 6.55, *p* = .011, η^2^ = .02; *F*_VR OE M_ (1, 299) = 9.59, *p* = .002, η^2^ = .03; *F*_VROE VO_ (1, 299) = 15.92, *p* < .001, η^2^ = .05), and as compared to the no-VR control condition (*F*_VROE NM_ (1, 299) = 9.20, *p* = .003, η^2^ = .03; *F*_VROE M_ (1, 299) = 12.92, *p* < .001, η^2^ = .04; *F*_VROE VO_ (1, 299) = 20.38, *p* < .001, η^2^ = .06). All other comparisons were not significant (*p*s > .10). Please see Table 1 for *n*, *M*, and *SD*s of all measures across the five conditions.

**Table 1:** Means (M) and Standard Deviations (SD) for spatial presence, inclusion of nature in self, connectedness to nature and donation to a pro-environmental organization, across conditions.

Condition	*n*	Spatial presence		Inclusion of nature in self		Connectedness to nature		Donation amount	
		*M*	*SD*	*M*	*SD*	*M*	*SD*	*M*	*SD*
VR OE voice-over	64	4.60	1.29	3.98	1.32	4.60	0.81	16.13	9.49
VR OE music	60	4.38	1.40	3.53	1.32	4.45	0.73	12.23	9.13
VR OE no- music	55	4.26	1.44	3.55	0.94	4.32	0.91	11.96	9.09
VR control	60	3.58	1.55	3.87	1.31	4.32	0.96	13.20	10.28
No-VR control	65	3.47	1.39	3.86	1.44	4.43	1.00	13.26	9.78

#### Connectedness to nature

For the first measure, Inclusion of Nature in the Self (INS), the one-way ANOVA revealed no main effect of condition on the extent in which participants’ included nature within their representation of self (*F*(4, 299) = 1.55, *p* = .189, η^2^ = .020). Follow up analyses showed that, according to the conventional *p* < .05 significance level, participants in the VR OE voice-over condition included nature not more in the self than in the VR OE no-music (*F*(1, 299) = 3.46, *p* = .064, η^2^ = .01), and the VR OE music conditions (*F*(1, 299) = 3.83, *p* = .051, η^2^ = .01). All other comparisons were not significant (*p*s > .10). The results of the one-way ANOVA for the second measure Connectedness to Nature Scale (CNS) also revealed no main effect of condition (*F*(4, 299) = 1.04, *p* = .389, η^2^ = .014). Follow up analyses showed that participants in the VR OE voice-over condition did not feel more connected to nature than in the VR OE no-music condition when applying the conventional *p* < .05 significance level (*F*(1, 299) = 2.94, *p* = .088, η^2^ = .01) and the VR control condition (*F*(1, 299) = 3.09, *p* = .080, η^2^ = .01). All other comparisons were not significant (*p*s > .10).

#### Donation amount

**Fig 2:**
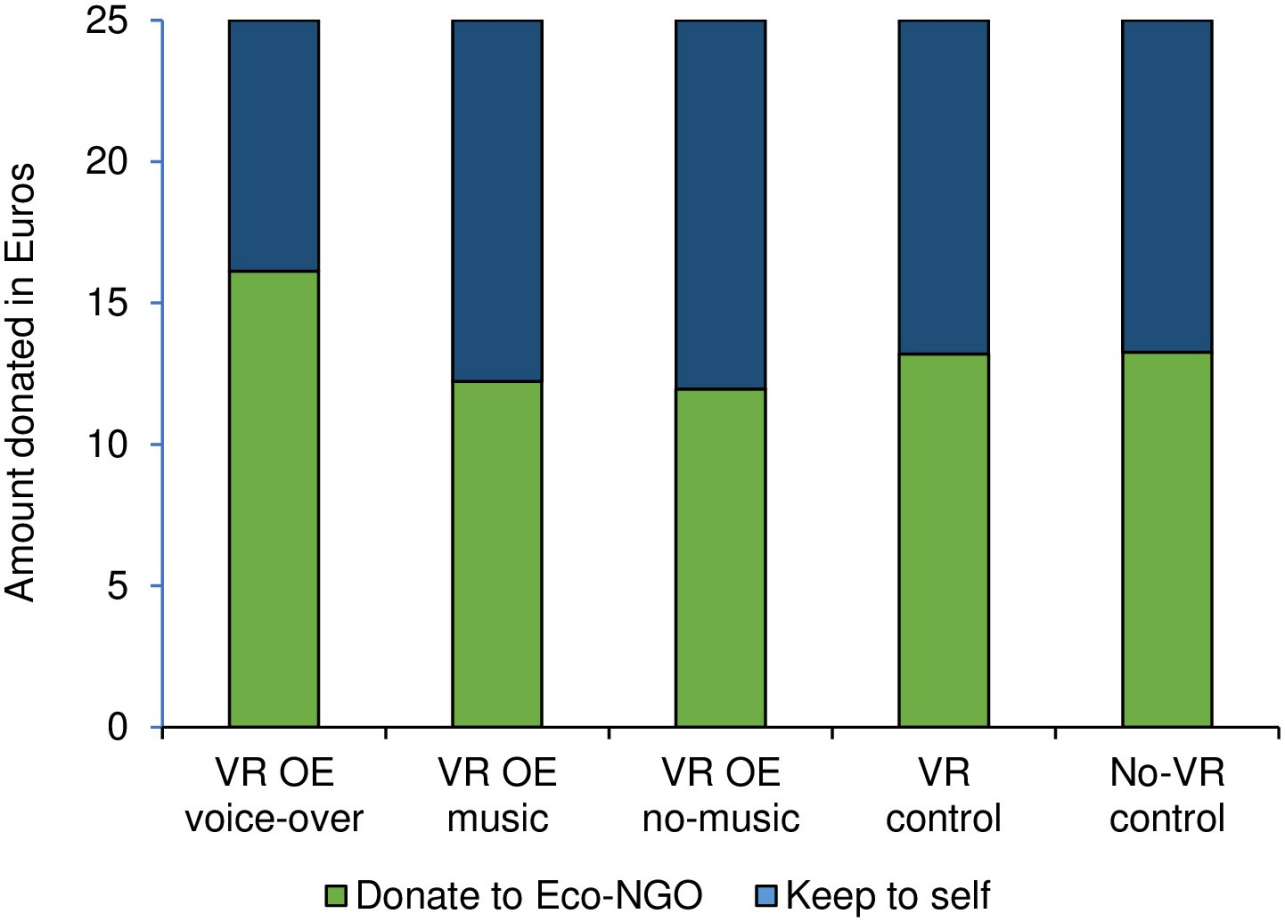
Amount donated to pro-environmental organization in Euros (as compared to keep to oneself).

To test whether the VR overview effect experience influenced participants’ willingness to donate to an environmental organization, a one-way ANOVA was conducted. The results revealed no main effect of condition (*F*(4, 299) = 1.84, *p* = .121, η^2^ = .02). Follow up analyses showed that participants in the VR OE voice-over condition donated significantly more money to an environmental organization compared to the VR OE no-music (*F*(1, 299) = 5.59, *p* = .019, η^2^ = .018) and the VR OE music conditions (*F*(1, 299) = 5.14, *p* = .024, η^2^ = .02). Despite the effects being directional, participants in the VR OE voice-over condition did not donate more money than in the VR control (*F*(1, 299) = 2.89, *p* = .090, η^2^ = .01) and the no-VR control conditions (*F*(1, 299) = 2.87, *p* = .091, η^2^ = .01; see Fig 2).

#### Mediation of inclusion with nature into self scale (INS) on donation amount

To test whether the extent in which connectedness to nature (INS) mediated the effect of condition on donation amount, a multicategorical mediation model was conducted (Model 4, 5000 bootstraps, with VR OE voice-over as baseline condition [42]). Results revealed that for all comparisons the indirect effects through Inclusion of Nature in Self were not significant (VR OE voice-over vs. VR OE music: β = -0.04, *se* = .03, 95% CI [-0.12, 0.00]; vs. VR OE no-music: β = -0.04, *se* = .03, 95% CI [-0.13, 0.01]; vs. VR control: β = -0.01, *se* = .03, 95% CI [-0.07, 0.03]; vs. no-VR control: β = -0.01, *se* = .03, 95% CI [-0.08, 0.04]), indicating that connectedness to nature did not mediate the effect.

#### Mediation of connectedness to nature scale (CNS) on donation amount

To test whether the extent in which connectedness to nature (CNS) mediated the effect of condition on donation amount, a multicategorical mediation model was conducted (Model 4, 5000 bootstraps, with VR OE voice-over as baseline condition [42]). Results revealed that for all comparisons the indirect effects through Inclusion of Nature in Self were not significant (VR OE voice-over vs. VR OE music: β = -0.07, *se* = .05, 95% CI [-0.18, 0.01]; vs. VR OE no-music: β = -0.04, *se* = .04, 95% CI [-0.13, 0.03]; vs. VR control: β = -0.07, *se* = .04, 95% CI [-0.17, 0.01]; vs. no-VR control: β = -0.05, *se* = .04, 95% CI [-0.15, 0.03]), indicating that connectedness to nature did not mediate the effect (See S8 Appendix in S1 File for exploratory mediation analyses of spatial presence).

#### Robustness check

Covariate analyses were conducted to check whether the extent in which participants felt cyber sick influenced any of the results. The covariate analyses revealed that cybersickness did not explain any of the variance for INS or donation task (*p*s > .10), but did for CNS (*F*(1, 298) = 4.54, *p* = .034, η^2^ = .02. However, the pattern of results of condition on the connectedness to nature scales (*F*_INS_(4, 298) = 1.54, *p* = .190, η^2^ = .02; *F*_CNS_ (4, 298) = 0.80, *p* = .529, η^2^ = .01) and the donation amount did not change (*F*(4, 298) = 1.82 *p* = .125, η^2^ = .02) after including cybersickness as covariate.

#### Subsequent pro-environmental behaviours: Daily meat and dairy consumption

To test whether the VR overview effect experience had an impact on participant’s subsequent pro-environmental consumption behaviour, two separate 5 (condition) X 3 (time: day 2, 4 and 6) repeated measures ANOVAs were conducted with meat consumption (How much meat did you eat today?) and dairy consumption (How much dairy did you consume today?) as dependent variables. Participants who did not fill out all three follow-up questionnaires or indicated that they never eat meat or dairy were excluded from the analyses, leaving a total *N* = 276 for meat- and *N* = 280 for dairy consumption (*n* = 60/61 VR OE voice-over; *n* = 56/57 VR OE music; *n* = 48/50 VR OE no-music; *n* = 54/55 VR control; *n* = 58/57 no-VR control).

**Fig 3:**
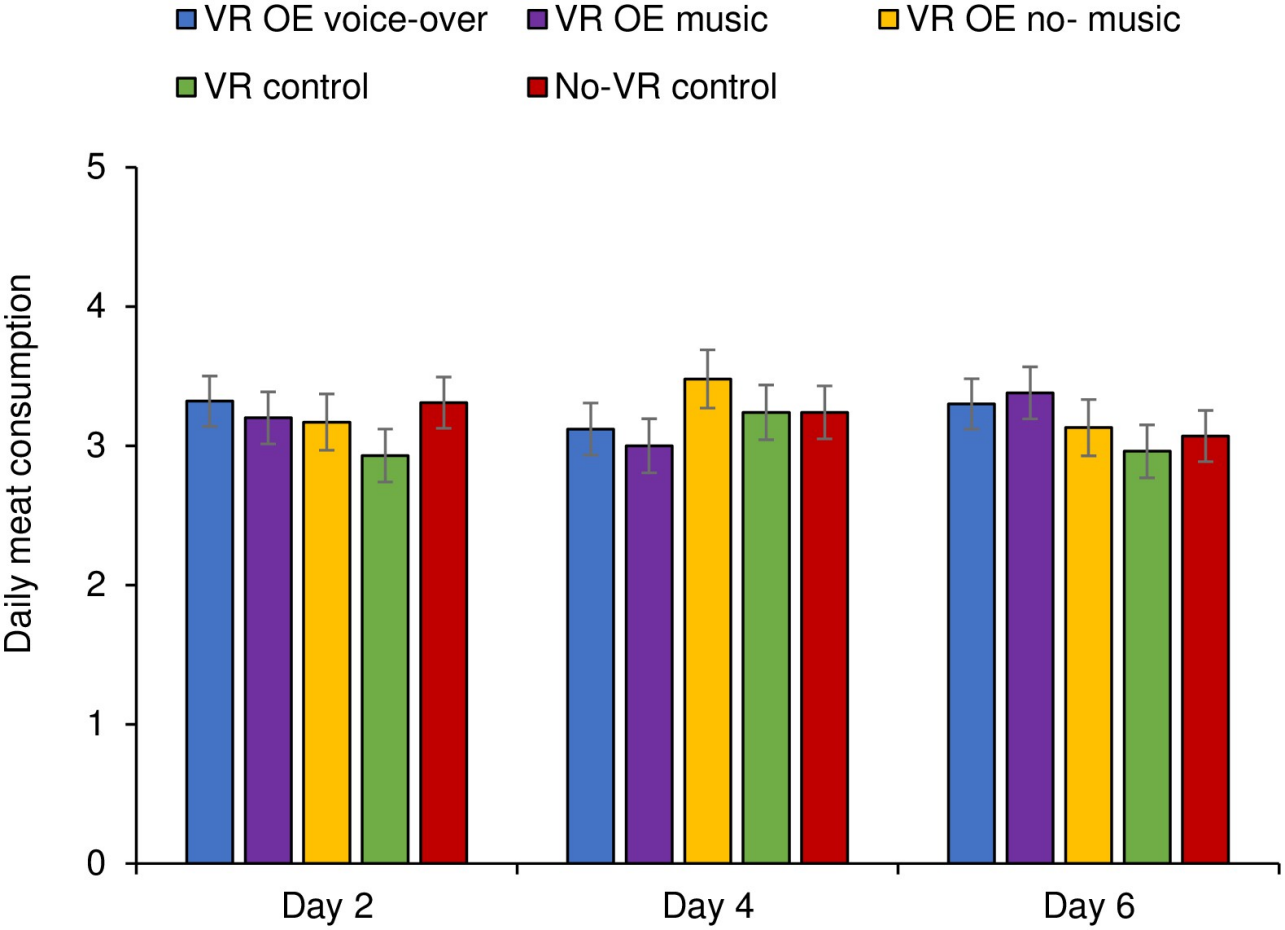
Daily meat consumption as a function of condition at day 2 (T1), day 4 (T1), and day 6 (T3).

A repeated-measures ANOVA showed that participants’ meat consumption did not differ across conditions (*F*(4, 271) = .30, *p* = .880, η_p_^2^ = .004), nor across time (*F*(2, 542) = 0.09, *p* = .915, η_p_^2^ < .001). The interaction between time and condition was not significant (*F*(8, 542) = 1.69, *p* = .098, η^2^ = .02; see Fig 3 and S6 Appendix in S1 File for all Means and SDs).

**Fig 4:**
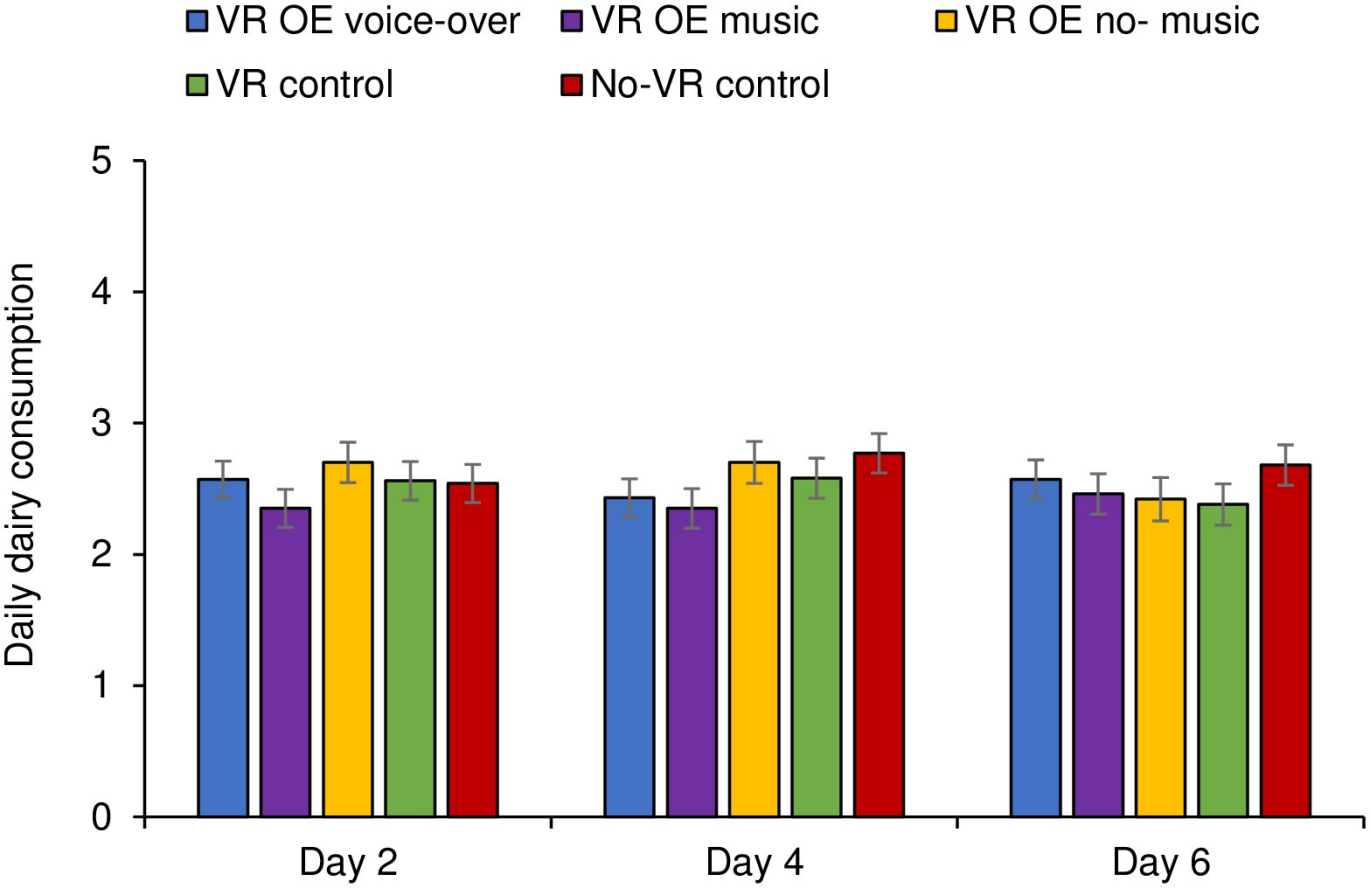
Daily dairy consumption as a function of condition at day 2 (T1), day 4 (T1), and day 6 (T3).

For dairy consumption, the results of the repeated-measures ANOVA also showed no main effect of condition (*F*(4, 275) = 0.70, *p* = .590, η_p_^2^ = .01), or of time (*F*(2, 550) = 0.37, *p* = .688, η_p_^2^ = .001). The interaction between time and condition was also not significant (*F*(8, 550) = 1.43, *p* = .183, η^2^ = .02; see Fig 4).

## General discussion

“I was not very active in fighting for our environment […] but with the spaceflight experience, this awareness was much more increased and I’m making much more conscious decisions now” (Franz Viehbock, Austria [11]). In the current research we examined whether experiencing the overview effect, like astronaut Viehbock expressed in the quote above, indeed promotes people’s pro-environmental behaviour. We used VR as a medium to create the experience of seeing the Earth from outer space and investigated its effect on short-term pro-environmental behaviours in the first study. In the second study, we used a longer VR experience of Earthgazing, systematically increased the level of technical immersiveness of the VR experience to investigate whether these changes would amplify the effect, and tested effects on longer lasting subsequent sustainable consumer behaviours. In both studies, we explored the mediating role of connectedness with Earth and nature. The findings of Study 1 showed that a short VR overview effect video did not significantly enhance connectedness with Earth, did not increase consumers’ intention to make more sustainable decisions, nor promote donations to a pro-environmental organization. As 7-minutes is a short experience, three 15-minute videos were created for Study 2 with different levels of technical immersiveness. Again, results revealed no significant overall effect of the conditions. Still, the most immersive experience (including meditative music and voice-over) seemed to be most effective with higher donation amounts to a pro-environmental organization and higher connectedness with nature than the no-music and only music VR overview effect videos. No effects were found for the subsequent meat and dairy consumption behaviours.

What may be the reasons that we did not find much evidence for the overview effect, especially in view of the anecdotal evidence by those who have been in space along with impressions of museum directors and the reported media? One reason may be that a virtual experience of the overview effect only leads to changes in behaviour that is more immediate and includes simpler changes to one’s personal life, such as donating or using recyclable shopping bags. Changing meat and dairy habits is a much more demanding task and perhaps less easy to be modified after a short VR experience in the lab [50]. In addition, the experience could yet not be sufficiently immersive or enduring for stronger effects to occur. Perhaps, the length of the video may play an important role to increase the effect of the overview effect experience. A recent study provides preliminary evidence showing that an overview effect video of 25 minutes indeed increased people’s connectedness to nature pre- and post the intervention. However, no differences between the VR overview effect experience and the control group were observed after the intervention [51].

Furthermore, the meditative narrative may have been a crucial determining factor. It is possible that especially a voice can make a difference by involving people more directly and by helping them to interpret the experience (what exactly do I see where?). In the current study, we only included a voice-over in one VR-condition, and this condition appeared to stand out (albeit not significantly in comparison to all other conditions) for one’s connectedness to nature and donation to a pro-environmental organization. By including a voice-over only condition, follow-up research could disentangle the individual effects of the overview effect with voice-over and voice-over only conditions.

A complementary reason may be that experiencing the overview effect is primarily effective when it is coupled with some type of integration practice such as social sharing. Rather than undergoing an individual experience in a laboratory, astronauts are likely to discuss the overwhelming experiences (e.g., the beauty of the globe, perhaps in combination with its fragility) with other astronauts during the space flight. Thereby the experiences themselves may be magnified, as well as their persistence over time. Such experiences may also be shared among people who visit museums together. Indeed, such effects of sharing emotions have been documented for a variety of phenomena, including emotion and social contagion in meetings (e.g., [52]), movies (e.g., laughter [53]), sports events (e.g., [54]), and climate change [55]. Thus, it is possible that the overview effect is more likely to materialize under social circumstances in which people can share observations and experiences.

How should one evaluate the empirical status of the overview effect? Clearly, anecdotal information is often based on small numbers. Also, it is possible that, after having been on spaceflights, astronauts may experience life on Earth as relatively “mundane” and therefore may become more strongly interested in contributing to the world. Thus, we cannot exclude the possibility that some anecdotal support is explained by coping with new situations rather than with mechanisms directly connected to the overview effect. Recent research demonstrated a positive effect of VR awe inspiring nature experience but not from an VR awe inspiring overview effect experience on pro-environmental behaviour [19]. However, in our view, it is too early to conclude that the overview effect is small or invalid. It is possible that other features, such as deeper immersion (i.e., longer video, undergoing the experience in a relaxing environment through receiving biofeedback), direct simulation of other astronaut experiences (i.e., simulation of weightlessness or beginning the space travel from home [14], or social sharing and discussion [56] are needed for a stronger and more persistent effect. Also, we cannot exclude the possibility that abstract information cannot be sufficiently captured by virtual reality, a tool that is often used to capture specific observations and social interactions (e.g., responses to various threats, such as spiders, or approaches to ethnic minorities [57, 58]).

### Limitations and future research

The use of virtual reality is often viewed as one of the most powerful tools to examine realistic observations under controlled experimental conditions. By focusing on short-term and subsequent longer term effects, along with potential mediating variables, we investigated the breadth of effects that one might expect from this experience. The fact that we examined various conditions of virtual reality has provided interesting insights, uncovering voice as one medium that may well be essential to the experience to be effective.

One limitation is however that we cannot tell whether voice alone or a voice-over guiding the virtual overview effect experience accounts for the effects, as the current design did not include a “narrative” only condition. Additionally, even though the results seem to indicate that length matters, as we observed directional effects in the study using a 15-minute video (but none in the 7-minute video study), the two studies differed in other respects as well, such as the information provided on the pamphlet before the experiment. Therefore no causal inferences regarding video length can be made. A research program that systematically investigates the length and level of technical immersiveness of the overview effect experience could establish more clearly if the overview effect experience is essential (or only a narrative would suffice) to influence pro-environmental behaviour, and if so, which features are crucially determining the effect.

The present findings do not directly demonstrate any behavioural change over substantial periods of time. Our work suggests some critical levels of immersion are needed to change (albeit directional) short-term behaviour (donations) or experiences (connectedness to nature). We may observe some change in short-term, rather than subsequent longer-term behaviours such as consuming meat and dairy, as they are less habit-driven. In addition, several mechanisms such as the experience of awe [18, 19] and a warm glow [59], might help explain why short-term, and not longer term behaviour, is affected. These short-term mechanisms, especially a warm glow, may result in a licencing effect preventing longer term behavioural change to occur [60]. The current study was not designed to provide a competitive test of these mechanisms, but instead to provide evidence whether aspects of the overview effect can occur and whether it requires a meaningful degree of immersion. The relative importance and strength of such mechanisms awaits future research.

Our study also adds to the growing body of literature on the effects of virtual reality on prosocial behaviour [61, 62], environmental behaviour change (e.g., [19, 36]), and on food changes specifically (e.g., [35, 63, 64]). Research for example shows that when seeing the impact of one’s food choices on the environment in VR, this affects people’s food choices in the following weeks due to an increase in personal response efficacy beliefs [35, 64]. For future research investigating the effect of the overview effect experience on longer term dietary preferences, we advise, in line with previous VR studies [35, 64], to add a clear personal (response) efficacy appeal to make the link between changing plant-based diet and alleviating climate change clearer.

## Concluding remarks

The overview effect has considerable appeal–the mere observation of the world from a distance may be such an overwhelming experience, that it facilitates concerns about the environment, climate change, and perhaps other threats to the health and vitality of our planet. Our findings tell a story of some modesty. The overview effect may well be conditional on specific circumstances, including the ways in which it is presented to us, providing new insights for businesses and public education campaigns, upon which future research can be build.

## Supporting information

S1 File (DOCX)

S1 Checklist *PLOS ONE* clinical studies checklist. (DOCX)
