# Supplementary material for: Observing the earth from space: Does a virtual reality overview effect experience increase pro-environmental behaviour?
Source: PLoS One. 2024 May 29;19(5):e0299883. doi: 10.1371/journal.pone.0299883 (PMC11135769; doi:10.1371/journal.pone.0299883)
Supplement: S1 File — (DOCX) [file pone.0299883.s001.docx]

**Supporting Information for “Observing the Earth from Space: Does a virtual reality overview effect experience increase pro-environmental behaviour?”**

**S1 Appendix. Pamphlet study 1**

**S2 Appendix. Measures and items studies 1 and 2**

**S3 Appendix. Correlations dependent variables study 1**

**S4 Appendix. Exploratory analyses study 1**

**S5 Appendix. Pamphlet study 2**

**S6 Appendix. Descriptive statistics study 2**

**S7 Appendix. Correlations dependent variables study 2**

**S8 Appendix. Exploratory analyses study 2**

**S1 Appendix. Pamphlet study 1**

**Planet Earth Has Its Limits**

Human actions on planet earth are changing the environment in an unsustainable direction. Factors contributing to this development have been categorised into the “nine planetary boundaries” by scientists. Each category has a certain threshold (a so-called *boundary*). Transgressing the boundaries increases the risk of driving Earth into an inhospitable state. Below, the nine planetary boundaries are explained in more detail. Please read the information attentively.

***Climate Change:***

This boundary has already been surpassed, through the rising temperature of earth’s atmosphere. Many consequences seem irreversible. Human actions, such as deforestation, continue to make it worse. Climate change can lead to sea level rise, extreme droughts, and precipitation, making planet earth less habitable.


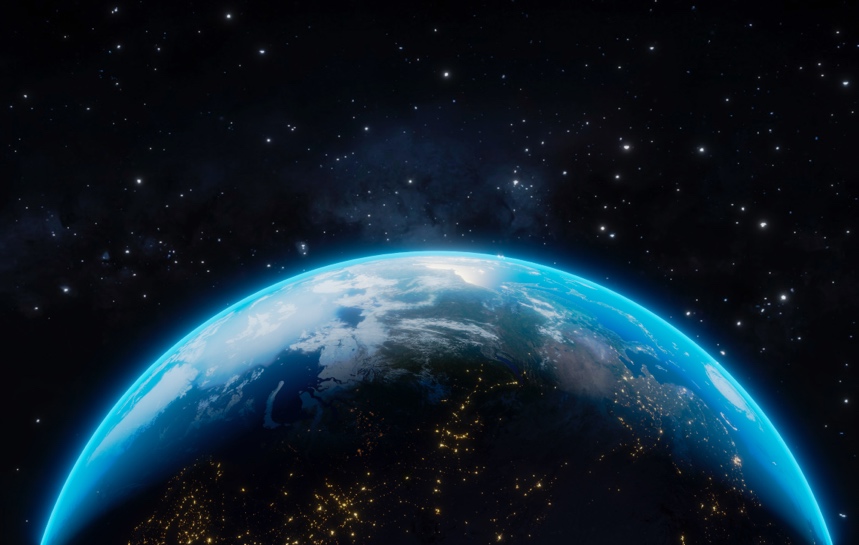


***Biodiversity Loss:***

Ecosystems are changing rapidly, risking the potential of irreversible changes. These changes have occurred rapidly over the past 50 years, due to the increased demand for food, water, and natural resources.

***Land System Change:***

Humans are continuously converting land into a utility. Forests, grasslands, and other types of land are being primarily converted to agricultural land. This land system change also widely impacts a range of other planetary boundaries, such as biodiversity loss or biogeochemical cycles.

***Freshwater Use:***

Water is becoming an increasingly scarce resource. This is caused by multiple factors, such as the increased human demand for water, land system change and climate change. Fresh water is vital for the survival of humans, as well as any animal and plant species.

***Biogeochemical Cycles:***

Many industrial and agricultural processes, such as fertiliser use, have radically changed the cycles of two very important elements: nitrogen and phosphorous. As too much nitrogen and phosphorous are used, the remnants end up in aquatic zones (e.g., sea, lakes). This leads to algae blooms which consume the oxygen in the water, not leaving enough oxygen for other species and turning the water bodies into “dead zones”.

***Ocean Acidification:***

A large proportion of the CO_2_ emitted into the atmosphere is dissolved into the oceans, transforming into carbonic acid. This acid alters the ocean’s pH level, causing negative effects on marine species. Much of the marine ecosystem struggles to grow in this more acidic environment, think for example of coral bleaching.

***Stratospheric Ozone Depletion:***

The stratospheric ozone layer in the atmosphere filters out ultraviolet (UV) radiation from the sun. If this layer depletes, a greater amount of UV radiation reaches earth, resulting in harmful consequences on earth’s living organisms. Fortunately, actions are being taken to not overstep this boundary.


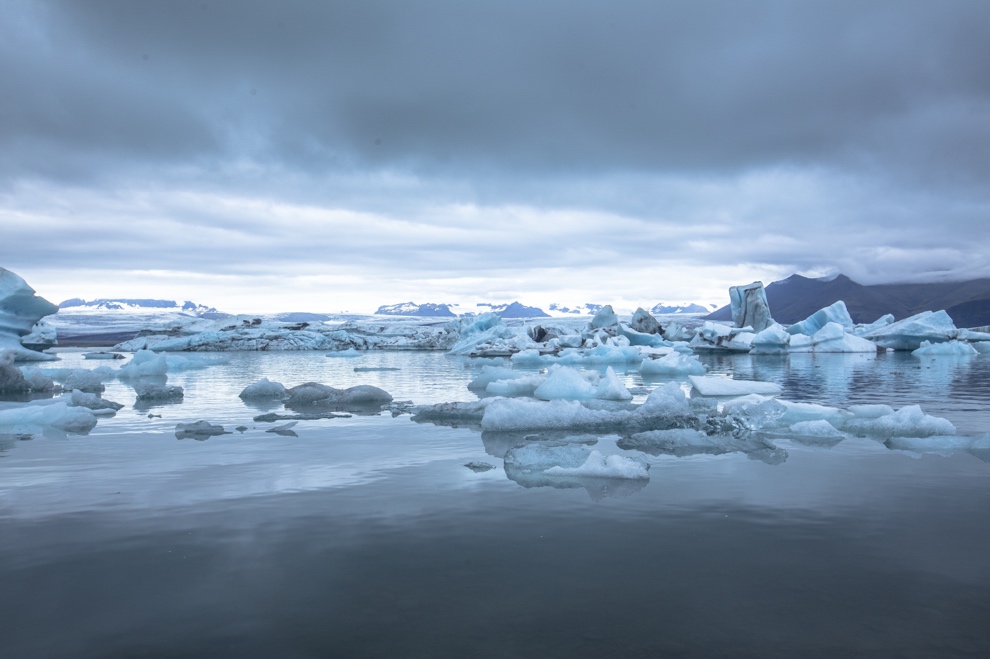


***Atmospheric Aerosol Pollution:***

Aerosols are small particles that play a critical role in a healthy atmospheric circulation, contributing to features such as cloud formation or radiation reflection. However, atmospheric pollution is negatively impacting the composition of these aerosols, leading to smog and an unhealthy air quality.

***Chemical Pollution:***

The industrial revolution has caused for a drastic increase in novel chemical pollution. These pollutants directly and indirectly negatively impact the health of earth’s organisms, due to toxicants that are being released.

Please note there are some slight differences between the pictures in the pamphlet used in the study and the pictures presented here due to copyright issues (designed by Freepik, [freepik.com](https://freepik.com/)).

**S2 Appendix. Measures and items studies 1 and 2**

| **Study 1** |
| --- |
| **Spatial Presence Experience Scale** (four items; Hartmann et al., 2016; α = .94) |
| (1) I felt like I was actually there in space; (2) It was as though my true location had shifted into space; (3) It felt as though I was physically present in space; (4) It seemed as though I was actually surrounded by space. |
| 1 = strongly disagree; 7 = strongly agree |
| **Dispositional Positive Emotion Scales** (six items; subscale from Shiota & John, 2006, α = .73) |
| (1) I often feel awe; (2) I see beauty all around me; (3) I feel wonder almost every day; (4) I often look for patterns in the objects around me; (5) have many opportunities to see the beauty of nature; (6) I seek out experiences that challenge my understanding of the world. |
| 1 = strongly disagree; 7 = strongly agree |
| **Study 2** |
| **Spatial Presence Experience Scale** (four items; Hartmann et al., 2016; α = .93) |
| Same as in Study 1 |
| **Connectedness to nature measure 2: Connectedness to Nature Scale** (thirteen items; Mayer & Franz, 2004; α = .85) |
| Right now, …  (1) I feel a sense of oneness with the natural world around me; (2) I think of the natural world as a community to which I belong; (3) I recognize and appreciate the intelligence of other living organisms; (4) I feel disconnected from nature (reverse coded); (5) When I think of my life, I see myself as part of a larger cyclical process of living; (6) I feel a kinship with animals and plants; (7) I feel as though I belong to the Earth as equally as it belongs to me; (8) I have a deep understanding of how my actions affect the natural world; (9) I feel part of the web of life; (10) I feel that all inhabitants of Earth, human, and nonhuman, share a common ‘life force’; (11) like a tree can be part of a forest, I feel embedded within the broader natural world; (12) I feel like I am only a small part of the natural world around me, and that I am no more important than the grass on the ground or the birds in the trees; (13) I feel my personal welfare is independent of the welfare of the natural world (reverse coded). |
| 1 = strongly disagree; 7 = strongly agree |
| **Commitment to the Environment Scale** (ten items; Davis et al., 2009; α = .88) |
| Right now, …  (1) I am interested in strengthening my connection to the environment; (2) I feel strongly linked to the environment; (3) I take into account how my decisions may affect the environment; (4) it seems to me that humans and the environment are interdependent; (5) it makes me feel good when something happens that benefits the environment; (6) it is important to me to feel a connection with the environment; (7) I feel that I will always have a strong connection with the environment; (8) I believe that the well-being of the natural environment can affect my own well-being; (9) I feel very attached to the natural environment; (10) I feel committed to keeping the best interests of the environment in mind. |
| 1 = strongly disagree; 7 = strongly agree |
| **Involvement in the Topic of Climate Change** (four items; Greussing, 2020; α = .86) |
| Right now, …  (1) the topic of climate change is interesting to me; (2) important to me; (3) useful to me; (4) something I would like to get more information about. |
| 1 = strongly disagree; 7 = strongly agree |
| **Environmental Concern scale** (five items; Ellis & Thompson, 1997; α = .77) |
| Right now I believe…  (1) that if things continue on their present course, we will soon experience a major ecological catastrophe; (2) that the problems of the environment are not as bad as most people think (reverse coded); (3) that the oceans are dying from oil pollution and dumping of waste; (4) that we are fast using up the world's natural resources; (5) that people worry too much about human progress harming the environment (reverse coded). |
| 1 = strongly disagree; 7 = strongly agree |
| **Cybersickness** (six items; Kennedy et al., 1993; α = .77) |
| To what extent did you feel the following sensations at any moment during this first task of the study?  (1) Fatigue; (2) Difficulty in focusing; (3) Dizziness; (4) Blurred vision; (5) Sweating; (6) Nausea. |
| 1 = strongly disagree; 7 = strongly agree |
| **Daily meat consumption (**measured on day 2, 4, and 6) |
| Please think back of your meals today (breakfast, lunch, dinner, in-between bites, and snacks).  How much meat (e.g., pork, beef, chicken, minced meat) did you eat today? |
| 1 (0 grams), 2 (1 – 75 grams, less than a deck of cards; e.g., a sandwich with meat topping), 3 (75–150 grams, as big as one deck of cards; e.g., a hamburger) , 4 (151–225 grams, two decks of cards; e.g., a hamburger and sandwiches with meat topping), 5(226–300 grams, three decks of cards; e.g., a hamburger, a chicken skewer, and sandwiches with meat topping), 6 (> 300 grams, more than three decks of cards; e.g., beef, croquette, and a hamburger),7 (I never eat meat) |
| **Daily dairy consumption (**measured on day 2, 4, and 6) |
| How much dairy (e.g., cow's milk, cream, yoghurt, cheese) did you consume today? |
| 1 (0 grams), 2 (1 – 200 grams, e.g., a glass of milk), 3 (201-400, e.g., a glass of milk and two buttered sandwiches with a cheese slice), 4 (401-600 grams, e.g., a carton of drink yoghurt), 5 (601-800 grams, e.g., a carton of drink yoghurt and two buttered sandwiches with a cheese slice), 6 (> 800 grams, e.g., a latte macchiato, a portion of oatmeal porridge, and a carton of drink yoghurt), 7 (I never consume dairy) |

*NB:* Items and measures included in the main manuscript are not included in the Table.

**S3 Appendix. Correlations dependent variables study 1**

**S3A. Correlations among dependent variables across conditions.**

| Variable | Presence | INS | Dispositional Awe | Donation | Shopping |
| --- | --- | --- | --- | --- | --- |
| Presence | 1.0 | - | - | - | - |
| INS | -.084 | 1.0 | - | - | - |
| Dispositional Awe | .105 | .312*** | 1.0 | - | - |
| Donation | -.094 | .098 | .053 | 1.0 | - |
| Shopping | -.025 | .253** | .045 | .109 | 1.0 |
| *Note.* * *p* < .05, ** *p* < .01, *** *p* <.001; INS = Inclusion of Nature in the Self scale | | | | | |

**S3B.** **Correlations among dependent variables in the VR Overview Effect condition.**

| Variable | Presence | INS | Dispositional Awe | Donation | Shopping |
| --- | --- | --- | --- | --- | --- |
| Presence | 1.0 | - | - | - | - |
| INS | .019 | 1.0 | - | - | - |
| Dispositional Awe | .335* | .366** | 1.0 | - | - |
| Donation | -.058 | .198 | .024 | 1.0 | - |
| Shopping | .098 | .419** | .054 | .199 | 1.0 |
| *Note.* * *p* < .05, ** *p* < .01, *** *p* <.001; INS = Inclusion of Nature in the Self scale | | | | | |

**S3C. Correlations among dependent variables in the VR Control condition.**

| Variable | Presence | INS | Dispositional Awe | Donation | Shopping |
| --- | --- | --- | --- | --- | --- |
| Presence | 1.0 | - | - | - | - |
| INS | -.197 | 1.0 | - | - | - |
| Dispositional Awe | .076 | .102 | 1.0 | - | - |
| Donation | -.091 | -.159 | -.041 | 1.0 | - |
| Shopping | -.034 | .164 | .085 | .060 | 1.0 |
| *Note.* * *p* < .05, ** *p* < .01, *** *p* <.001; INS = Inclusion of Nature in the Self scale | | | | | |

**S3D. Correlations among dependent variables in the no-VR Control condition.**

| Variable | Presence | INS | Dispositional Awe | Donation | Shopping |
| --- | --- | --- | --- | --- | --- |
| Presence | 1.0 | - | - | - | - |
| INS | -.082 | 1.0 | - | - | - |
| Dispositional Awe | -.012 | .513*** | 1.0 | - | - |
| Donation | -.180 | .221 | .185 | 1.0 | - |
| Shopping | -.104 | .118 | -.030 | .045 | 1.0 |
| *Note.* * *p* < .05, ** *p* < .01, *** *p* <.001; INS = Inclusion of Nature in the Self scale | | | | | |

**S4 Appendix. Exploratory analyses study 1**

For exploratory reasons we investigated the effect of the Overview Effect experience on *dispositional* awe and whether *dispositional* awe moderated the effect of condition on any of the three DVs, as dispositional awe was significantly correlated with the connectedness to nature measure (see WA C). The one-way ANOVA showed no differences of condition on dispositional awe (*F*(2, 142) = .33, *p* = .721, η^2^ = .005). We then conducted a moderation analysis with dispositional awe as a continuous variable (Process, model 1) to assess whether dispositional awe moderated the effects of condition on the three DVs. First, to test the effect on connectedness to nature, we included condition (dummy coded with VR Overview Effect condition (VR OE) as baseline) and dispositional awe (mean averaged) as predictors in the model. The results of the regression analyses demonstrated a significant effect of dispositional awe on connectedness with nature (*b* = 0.61, *t* = 2.97, *p* = .004, 95% CI [0.20, 1.02]) and a marginally significant interaction between VR OE vs. VR Control and dispositional awe (*b* = -0.47, *t* = -1.70, *p* = .092, 95% CI [-1.03, 0.08]), but no interaction between VR OE vs. no-VR Control and dispositional awe (*b* = 0.18, *t* = 0.57, *p* = .569, 95% CI [-0.44, 0.79]). When we examined the associations of awe with connectedness with nature across the three conditions, the results showed that the association of dispositional awe was significant in both the VR OE (*b* = 0.61, *t* = 2.75, *p* = .008, 95% CI [0.17, 1.06]) and the no-VR control condition (*b* = 0.79, *t* = 3.96, *p* < .001, 95% CI [0.39, 1.19]), but this association was not significant in the VR C condition (*b* = 0.14, *t* = 0.70, *p* = .488, 95% CI [-0.26, 0.53]). These results indicate that when people were high in their disposition to experience awe, they were only more likely to include earth into their representation of self in both the VR overview effect condition and in the no-VR control condition. This was however not the case for the VR control condition. Awe did not moderate the effect of condition on the shopping task nor the donation task (all *p*s > .10).

**S5 Appendix. Pamphlet study 2**

**Planet Earth Has Its Limits**

Human actions on planet earth are changing the environment in an unsustainable direction. Factors contributing to this development have been categorised into the “planetary boundaries” by scientists. Some of these boundaries are climate change, biodiversity loss, land system change, and freshwater use. Each category has a certain threshold (a so-called *boundary*). Transgressing the boundaries increases the risk of making planet earth less habitable. What can we do to reverse the direction? Please read the information below attentively.


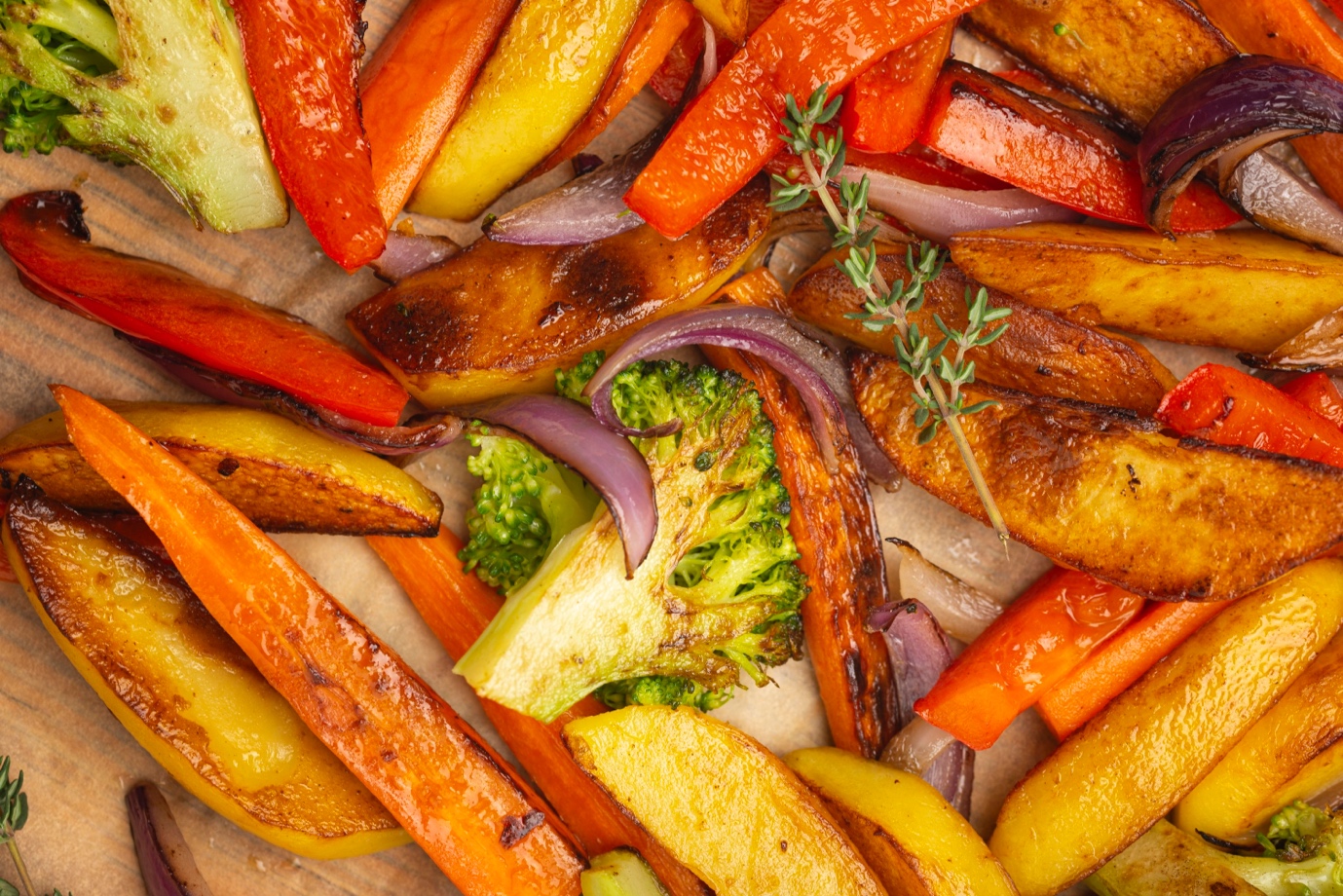


 
**What Can We Do?**

The global climate is changing because of the actions of humans. To stop or reverse this change, large-scale changes in consumption behaviours are necessary.

2 minute read
BY **Austa somichian-clausen**

PUBLISHED APRIL 18, 2022

**Consuming less meat** and dairy is one of the most impactful ways of reducing emissions that is readily available to individuals.

Each puff of methane coming out of a cow’s “plumbing,” added together, can have a big effect on the climate. Methane is an extremely potent greenhouse gas that causes damage much quicker than others, such as carbon dioxide (CO2).

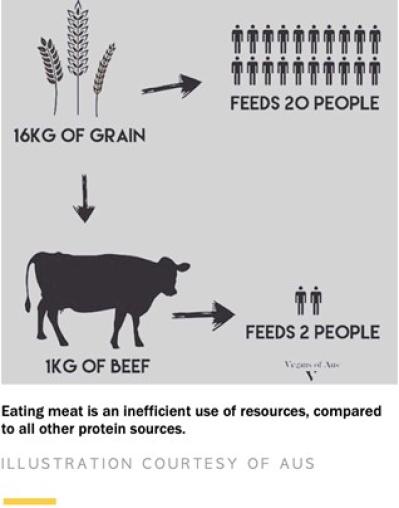
This potent nature of methane makes reducing numbers of farm animals a particularly attractive target, given that we desperately need to cut greenhouse gas emissions as soon as possible to avoid the worst impacts of the climate crisis.

Eating plants is also a far more efficient use of the planet’s stretched resources than feeding the plants to animals and then eating them. The global livestock herd and the grain it consumes takes up 83% of global farmland but produces just 18% of food calories.

The evidence is clear: if you want to have a sustainable diet, it is going to have much less red meat and dairy than today’s standard western diets.

This change does not have to be hard, as nowadays plant-based alternatives are abundant in supermarkets and in restaurants. Oat milk, for instance, can be used in all recipes that call for milk.

Changing your diet to be more eco-friendly by consuming less meat and dairy products is one of the best things you can do to combat climate change.

Please note there are some slight differences between the pictures in the pamphlet used in the study and the pictures presented here due to copyright issues (designed by Freepik, [freepik.com](https://freepik.com/)).

**S6 Appendix. Descriptive statistics study 2**

**S6A. Means (M) and Standard Deviations (SD) for Commitment to the Environment, Involvement in the Topic of Climate Change, and Environmental Concern, per condition.**

| Condition | *n* | Commitment to the Environment | | Involvement in the Topic of Climate Change | | Environmental Concern | |
| --- | --- | --- | --- | --- | --- | --- | --- |
|  |  | *M* | *SD* | *M* | *SD* | *M* | *SD* |
| VR OE voice-over | 64 | 4.74 | 0.99 | 5.16 | 0.97 | 5.46 | 0.99 |
| VR OE music | 60 | 4.51 | 1.00 | 4.86 | 1.02 | 5.38 | 0.99 |
| VR OE  no-music | 55 | 4.51 | 0.99 | 4.81 | 1.31 | 5.33 | 1.07 |
| VR control | 60 | 4.64 | 1.00 | 5.26 | 1.03 | 5.54 | 0.94 |
| No-VR control | 65 | 4.58 | 1.01 | 5.03 | 1.22 | 5.48 | 0.95 |

**S6B. Means (M) and Standard Deviations (SD) for meat and dairy consumption on Day 2 (T1), Day 4 (T2), Day 6 (T3), per condition**

|  | Meat consumption | | | | | | | Dairy consumption | | | | | | |
| --- | --- | --- | --- | --- | --- | --- | --- | --- | --- | --- | --- | --- | --- | --- |
| Condition | *n* | Day 2 | | Day 4 | | Day 6 | | *n* | Day 2 | | Day 4 | | Day 6 | |
|  |  | *M* | *SD* | *M* | *SD* | *M* | *SD* |  | *M* | *SD* | *M* | *SD* | *M* | *SD* |
| VR OE voice-over | 60 | 3.32 | 1.26 | 3.12 | 60 | 3.30 | 1.34 | 61 | 2.57 | 1.09 | 2.43 | 1.07 | 2.57 | 1.16 |
| VR OE music | 56 | 3.20 | 1.37 | 3.00 | 56 | 3.38 | 1.34 | 57 | 2.35 | .94 | 2.35 | 1.14 | 2.46 | 1.15 |
| VR OE no-music | 48 | 3.17 | 1.53 | 3.48 | 48 | 3.13 | 1.48 | 50 | 2.70 | 1.28 | 2.70 | 1.23 | 2.42 | 1.18 |
| VR control | 54 | 2.93 | 1.41 | 3.24 | 54 | 2.96 | 1.52 | 55 | 2.56 | 1.07 | 2.58 | 0.98 | 2.38 | 1.16 |
| No-VR control | 58 | 3.31 | 1.44 | 3.24 | 58 | 3.07 | 1.32 | 57 | 2.54 | 1.09 | 2.77 | 1.24 | 2.68 | 1.17 |

**S7 Appendix. Correlations dependent variables study 2**

|  | Connectedness to nature | IES | Donation amount | Day 2 Meat Consumption | Day 2 Dairy Consumption |
| --- | --- | --- | --- | --- | --- |
| Connectedness to nature | 1.0 | - | - | - | - |
| INS | .470* | 1.0 | - | - | - |
| Donation amount | .244* | .135* | 1.0 | - | - |
| T1 Meat Consumption | -.098 | -.034 | -.092 | 1.0 | - |
| T1 Dairy Consumption | -.214* | .041 | -.167* | .183* | 1.0 |

*Note.* * = Values significant at the .05 level. INS = Inclusion of Nature in Self scale

**S8 Appendix. Exploratory analyses study 2**

Exploratory, we tested whether spatial presence mediated the effect of condition on donation amount using the same multicategorical mediation model (Model 4, 5000 bootstraps, with VR OE voice-over as baseline condition, Hayes, 2017). Results showed first that participants in the VR OE voice-over condition felt more spatial presence than in both control conditions (*b* = -1.01, *se* = 0.25, *p* < .001, 95%CI [-1.51, -0.51]; *b* = -1.12, *se* = 0.25, *p* < .001, 95%CI [-1.61, -0.63]; respectively), but not more than the VR OE music and no-music conditions (*ps* > .10). Furthermore, when included into the model, spatial presence had a positive effect on donation amount (*b* = 0.88, *se* = 0.39, *p* = .025, 95%CI [0.11, 1.64]). Importantly, the indirect effect through spatial presence was significant for VR OE voice-over vs. VR control (*b* = -0.88, *se* = .45, 95%CI [-1.87, -0.12]); vs. no-VR control (*b* = -0.99, *se* = .49, 95%CI [-2.05, -0.12]), whereas the difference between VR OE voice-over and VR OE music/VR OE no-music in donation amount were both not mediated by spatial presence (indirect effect voice-over vs. music (*b* = -0.18, *se* = .25, 95%CI [-0.80, 0.19]) and vs. no-music (*b* = -0.30, *se* = .28, 95%CI [-0.97, 0.10])). These results indicate that the significant difference between VR OE voice-over and the two control conditions in donation amount was driven by perceptions of spatial presence, whereas the difference in donation between the VR OE voice-over and VR OE music and no-music conditions were not due to perceptions of spatial presence.
